# Pricing and Quantity Decisions under Asymmetric Carbon Emission Reduction Information and Cap-and-Trade Mechanism

**DOI:** 10.3390/ijerph20031944

**Published:** 2023-01-20

**Authors:** Faqi Xie, Yushuang Deng, Huiru Chen

**Affiliations:** 1 School of Business Administration, Chongqing Technology and Business University, Chongqing 400067, China; 2 College of Mathematics and Statistics, Huanggang Normal University, Huanggang 438000, China

**Keywords:** cap-and-trade, asymmetric information, emission reduction, signaling game

## Abstract

With the continuous spread of cap-and-trade mechanisms and consumers’ great concerns about environmental issues, manufacturers strive to reduce carbon emissions. Unfortunately, they are not always willing to disclose their accurate emission reductions or may even falsify emission reduction information. By developing a signaling model, we explore the impact of the cap-and-trade mechanism and asymmetric information on the decision-making of supply chain members composed of a manufacturer regulated by the cap-and-trade mechanism, and a retailer. As a result, we show that the low-type manufacturer has the incentive to mimic the pricing behavior of the high-type manufacturer under information asymmetry. Thus, in order to avoid this mimicry, the high-type manufacturer will distort the wholesale price. Moreover, the impact of the cap-and-trade mechanism on the manufacturer depends on the initial quotas. Only when the initial quota is in the middle range does the high-type manufacturer benefit, while the low-type manufacturer suffers. Furthermore, the low-type manufacturer tends to hide emission reduction information, while the high-type manufacturer tends to disclose the information. We also address how information asymmetry weakens the emission reduction advantages of the high-type manufacturer. However, the cap-and-trade mechanism can effectively alleviate this negative effect.

## 1. Introduction

As global warming poses a more serious threat to human society, the world community is actively taking measures to reduce carbon emissions to delay the intensification of global warming. Among various policies and regulations, the cap-and-trade mechanism is a widely-used and effective measure [1,2,3]. The cap-and-trade mechanism is based on the 1997 Kyoto Protocol, which first proposed that carbon dioxide emission rights could be traded like ordinary commodities. After years of development, many countries and regions around the world are applying the cap-and-trade mechanism, such as Britain, Australia, Japan, and so on. The European Union Emission Trading System (EU-ETS) is at the forefront of the world. Coincidentally, the Chinese government began to launch the China Carbon Emission Trade Exchange (CCETE). The pilot project was started in 2013 and gradually promoted nationwide in 2017. During the pilot period, carbon emissions were reduced by 129.588 million tons [4].

The key to the cap-and-trade mechanism is to use market means to control carbon emissions in two steps. First, the government allocates the initial carbon emission quotas to companies [5]. The total amount of the initial carbon quotas for allocation, directly, is the maximum carbon emission of regulated companies within a specified period of time. Next, a company can freely trade carbon quotas in the carbon trading market [6]. Due to changes in emission reduction technology and economic activity level, the actual carbon emissions are usually different from the allocated initial carbon quotas. If the actual carbon emissions are greater than the initial carbon emission quota, they need to buy quotas so that excess carbon emissions can be legally discharged. Conversely, if the quotas are surplus, companies can sell the additional parts. Thus, when the initial carbon emission quotas become tightened slightly every year, the carbon trading price becomes higher which is determined by the carbon trading market. So, the cap-and-trade mechanism can impel companies to constantly innovate to reduce carbon emissions [7,8].

Moreover, the public universally follows environmental issues with interest and favors low-carbon products. As consumers are also aware of the importance of environmental protection, low-carbon products have become a popular choice. For example, more and more Chinese consumers are willing to buy environment-friendly electric vehicles; 67% of Americans believe that the emergence of green products has affected their purchase decisions; the proportion of European consumers who prefer low carbon products has increased significantly from 31% in 2005 to 75% [9]. This preference for low-carbon products translates into purchasing power, which means more and more consumers are willing to spend more on low-carbon products [10,11]. Recently, according to a survey of public environmental awareness, nearly 70% to 80% of the respondents are willing to pay for environmental improvement and the purchase of environmental protection products [12].

Manufacturers pay more and more attention to carbon emission reduction, but their emission reduction level is not often announced to the public. Manufacturers invest in technology and capital to reduce carbon emissions in the production process, in response to the appeal of the cap-and-trade mechanism and the market for environmental protection [13,14]. For example, Baosteel Group Corporation, the largest iron and steel conglomerate in China, is actively developing hydrogen metallurgy technology to replace coke in the traditional process, so as to greatly reduce emissions from the iron-making process [15]. However, there is a problem in that the emission reduction level is usually privately grasped only by the manufacturers. Intuitively, manufacturers are reluctant to disclose carbon information. Manufacturers would voluntarily participate in the CDP and publish reports such as sustainable development reports to disclose carbon information. However, because of the non-standard computing system and imperfect information sharing mechanism, the quality and reliability of the sustainable development reports disclosed by enterprises cannot be guaranteed [16]. Some enterprises will even carry out greenwashing practices, which means that enterprises that are destructive to the environment will manipulate the information disclosure and create the illusion of environmental friendliness, which will challenge the credibility of the information disclosure [17]. These manufacturers, who are regulated by cap-and-trade regulations, sell their low-carbon products through retailers. For example, the products produced by Baosteel Group Corporation need to be sold through sales agencies all over the country. Since it is impossible to ensure that the manufacturer publishes the carbon emission efficiency truly and accurately, there is carbon emission reduction information asymmetry between the retailer and manufacturer. Thus, on account of the above considerations, the following key questions are worth discussing: (1) How will the manufacturer and retailer determine optimal decisions to maximize their own profits with asymmetric information on carbon emission reduction? (2) What is the impact of the cap-and-trade mechanism on the performances of the supply chain members? (3) How does the asymmetry of carbon emission reduction information affect the performance of supply chain members?

In order to address the aforementioned questions, we construct a framework composed of a manufacturer who is subjected to the cap-and-trade mechanism, and a retailer. There are three scenarios considered in our study that capture three stages in the process of policy implementation: (1) original scenario, in which the cap-and-trade mechanism is not implemented and the manufacturer privately has carbon emission reduction information; (2) implementation scenario, where the cap-and-trade mechanism is put into effect but information is still asymmetric; (3) transparency scenario, in which the cap-and-trade mechanism continues to be implemented, but the information becomes transparent. Under information asymmetry, the manufacturer acts as the signal sender and uses the wholesale price to transmit emission reduction information to the retailer, who is the signal receiver. Then, we compare the equilibrium in three scenarios to investigate the effects of the cap-and-trade mechanism and asymmetric information. Additionally, we analyze the effect of consumer environmental awareness and carbon trading price on the equilibrium results through sensitivity analysis. We have made some conclusions as seen below.

First, regarding the cap-and-trade mechanism, we find that the cap-and-trade mechanism can effectively urge the manufacturer to reduce emissions, only when the initial quota is in a relatively middle area. This area is determined by the manufacturer’s endogenous technological uncertainty and does not depend on the traditional initial quota allocation factors: historical emissions or industry benchmarks. In this area, the high-type manufacturer is better off, while the low-type manufacturer is worse off compared with no cap-and-trade mechanism. However, the cap-and-trade mechanism is always harmful to the retailer. Furthermore, the initial quota and carbon trading price work together to affect the changing trend of manufacturers’ profits. When the initial quota is large, the manufacturer’s optimal profit increases with the carbon trading price, and vice versa. However, the retailer’s profit always decreases with the carbon trading price.

Second, regarding asymmetric information, it is not always beneficial for manufacturers to have private information on emission reduction rates. Under information asymmetry, the low-type manufacturer is motivated to mimic the pricing behavior of the high-type manufacturer, which can induce the retailer to believe that the low-type manufacturer has high carbon emission reduction. Thus, the high-type manufacturer will distort the wholesale price to avoid this mimicry. This distortion results in a loss of profits. Conversely, whether the information is symmetric or not, the performance of the low-type manufacturer is the same. Moreover, information asymmetry also weakens the emission reduction advantages of the high-type manufacturer. However, the cap-and-trade mechanism can effectively alleviate this negative effect.

Third, regarding consumer environmental awareness, the improvement in the consumer environmental awareness is beneficial to all supply chain members. As the consumers’ environmental awareness increases under asymmetric information, the optimal wholesale price, determined by the high-type manufacturer, will decrease first and then increase.

The remainder of the paper is organized as follows. In the next section, we review the literature closely related to this research. This is followed by some basic assumptions and our model setting in Section 3. Section 4 and Section 5 investigate the equilibrium solutions under three scenarios. In Section 6, we analyze the impact of the cap-and-trade mechanism, asymmetric information, and the consumer environmental awareness on supply chain members. This study ends with conclusions and some directions for future research in Section 7. To enhance the readability, all proofs are placed in the Appendices Appendix A and Appendix B.

## 2. Literature Review

In this section, we review the literature closely related to this research, which is mainly divided into two main research streams: the cap-and-trade mechanism and asymmetric information.

### 2.1. The Cap-and-Trade Mechanism

The first stream of research focuses on investigating the cap-and-trade mechanism in supply chain management. Some scholars consider the problems of the emission reduction investment strategy [18,19,20] and inventory decisions [21,22,23,24]. In addition, some scholars analyze how the cap-and-trade mechanism affects production and management decisions from a different perspective, which is the main field of our research. On the one hand, some literature study the cap-and-trade mechanism under the production of two kinds of green products and non-green products. Gong and Zhou [25] investigate the production problem of the manufacturer with both green technology and regular production technology, and present that the initial quota and carbon trading price will determine the two technology options. Toptal et al. [1] study the optimization problem when the manufacturer sells the ordinary and the low-carbon product to consumers with a low-carbon premium, and they note that the carbon trading price will reduce the gap between the two products. On the other hand, some literature study the management problems when the manufacturer only produces green products. Xu et al. [26] investigate the impact of emission trading price on production and pricing in a make-to-order supply chain, and conclude that the emission trading decisions follow a two-threshold policy. Xia et al. [27] consider social preference, which relaxes the assumption that supply chain members are rational, and they study the impact of social preference and low-carbon awareness on supply chain members’ decision-making and performance. Chai et al. [28] analyze the impact of the cap-and-trade mechanism in re-manufacturing, and find that the carbon trading price plays a greater role in reducing emissions and improving profits than the initial carbon quota. Liu et al. [29] consider a supply chain with two competitive manufacturers, and show that consumers’ low carbon preference and carbon trading price have an important impact on manufacturers’ decision-making. Tang and Yang [30] analyze supply chain members with different capital adequacy, and find that the increase in the initial carbon quota and carbon trading price can effectively improve social welfare.

The above literature mainly believes that all supply chains can obtain the specific emission reduction level from manufacturers. As a result of the manufacturer’s internal decision, emission reduction is not always disclosed to the public. Thus, in order to make it more realistic, this study introduces asymmetric information into the cap-and-trade mechanism, which helps provide valuable insights for application of the cap-and-trade mechanism. On this account, we propose an incomplete information dynamic game that the emission reduction is the manufacturer’s private information. Different from previous studies, we find the range of initial carbon quotas that can effectively stimulate the internal motivation of the manufacturer to reduce emissions under asymmetric information, which can damage the profits of the relative low emission reduction manufacturer, and improve the emission reduction level.

### 2.2. Asymmetric Information

The second stream of research focuses on studying asymmetric information. Previous scholars discuss a variety of information asymmetry, mainly including widespread asymmetric information and distinctive asymmetric information. Widespread asymmetric information has attracted much attention in the field of supply chain management, such as demand information [31,32,33,34], cost information [35,36,37], quality information [3,38,39], and so on. It is also so abundant in the green supply chain. Hong et al. [40] study the effect of asymmetric green production cost information on product pricing strategy when there is competition between green products and non-green products in the market. They find that the green manufacturer needs to raise prices when asymmetric green production costs are high. Zhou et al. [41] analyze the manufacturer’s production decision when the retailer with fair concerns has private demand information, and they find that the manufacturer always chooses to produce green products if they have a choice between green products and non-green products. Xia and Niu [42] investigate how manufacturers deal with the problem that only the retailer has market information when both the manufacturer and retailer make efforts to reduce carbon emissions in a two-echelon supply chain. Additionally, they show that the manufacturer can induce the retailer to disclose market information, and actively participate in emission reduction activities through a series of contracts.

Moreover, distinctive asymmetric information only exists in carbon emission reduction. Xia and Niu [43] analyze that the government designed carbon contractual policy to encourage firms to reduce emissions, and consider two kinds of information asymmetry of carbon emission efforts and costs. They reveal that the carbon contract menu can induce the firm to reveal its real carbon-reducing capacity and encourage emission reduction. Li et al. [44] construct a supply chain consisting of the manufacturer and supplier where abatement costs and efforts of the manufacturer are private for the government. Additionally, they find that the government should consider the types of manufacturers when formulating linear incentive contracts. Ma et al. [45] consider the impact of government subsidies on enterprise performance under asymmetric emission reduction information. They find that the subsidy policy can encourage the manufacturer to actively share information if the subsidy can effectively increase demand. Wang et al. [46] analyze the impact of emission reduction efficiency on supply chain performance in the supply chain of a product designer and a manufacturer. Zhang et al. [47] explore the contract design problem when the supplier carries out environmental innovation, but the environmental innovation efficiency is not published to the retailer. They find that the retailer can use two kinds of contract, including a two-part tariff contract and an innovation effort requirement contract, to incite the manufacturer to reveal the information. Moreover, some scholars also study the problem of information asymmetry under the cap-and-trade mechanism. Yuan et al. [48] consider a supply chain composed of a retailer and a manufacturer, who has private emission reduction information and goes along with cap-and-trade mechanism, and investigate the problem of designing an incentive contract to encourage the disclosure of carbon emission information. Ma et al. [49] consider the supply chain of a manufacturer, subject to the cap-and-trade mechanism and multiple suppliers, where the supplier holds private information about the unit emission rate of the raw materials. In addition, they determine an effective carbon emission trading policy to control manufacturers’ carbon emissions. Li et al. [17] studied the information asymmetry between the manufacturer and consumers, where low-carbon preference is private for consumers, and they built three abatement mechanisms. Additionally, they found that the information rent caused by information asymmetry affects the efficiency of emission reduction.

We make a comparison between the above literature and our paper, as shown in Table 1. At present, asymmetric information in emission reduction activities is focused on carbon reduction efficiency. When the cap-and-trade mechanism is considered, the information asymmetry of carbon emission efficiency is often more directly related to this mechanism. That is exactly what we do. In addition, most studies use the mechanism design method to analyze the problem of information asymmetry, but we use the signal game method, which is consistent with the method adopted by Ma et al. [45]. What differentiates our model from theirs includes two aspects: one is that, as mentioned above, we consider the cap-and-trade mechanism in our model while they consider government subsidy, and the other is that we consider a complete supply chain while they consider only one enterprise. By building a signaling game model in which the manufacturer is the signal sender and the retailer is the signal receiver, we find that the counter intuitive thing is that the h-type manufacturer will set a lower wholesale price under asymmetric information to hurt his profit.

## 3. Model

Consider a low-carbon product supply chain including two members, a retailer (she) and a manufacturer (he) who is subject to the cap-and-trade mechanism. In order to deal with this mechanism, the manufacturer utilizes carbon emission reduction technology to produce low-carbon products. Then, he sells these products at a wholesale price *w* to the retailer, who orders in quantity *q* and sells them to consumers with a preference for low-carbon products. In the subsequent analysis, *m* and *r* are denoted as the manufacturer and retailer, respectively.

The supply chain members have asymmetric information about the carbon emission reduction rate. The emission reduction rate is an effective indicator that can reflect the outcome of emission reduction technology. As long as the manufacturer does not actively disclose the emission reduction rate, it is difficult for the retailer to obtain the accurate emission reduction rate. We assume that the carbon emission reduction rate $\tilde{e}$ of the manufacturer is ex ante random, and exhibits two values: either high $e_{h}$ with probability μ or low $e_{l}$ with probability 1−μ, where $1\geq e_{h}>e_{l}\geq 0$. Thus, the retailer only learns about the prior distribution of emission reduction rate when she orders from the manufacturer. On the contrary, the actual emission reduction rate is the manufacturer’s private information, which means that it is only in his own hands. Thus, there are two types i∈{l,h} of manufacturer according to the emission reduction rate. For example, the sales agent of Hebei Iron&Steel Group does not know the specific emission reduction, while Hebei Iron&Steel Group has a clear understanding of the whole product production process, including the emission reduction rate. Moreover, focusing on short-term decisions, we do not put long-term emission reduction investment into consideration and we normalize that the cost of emission reduction is zero.

As one of the driving forces of the manufacturer’s emission reduction, the cap-and-trade mechanism is implemented in two steps. First, a “cap” is allocated. There is a ceiling (i.e., cap) on carbon emissions, which will be tightened slightly every year, so the total emissions will decrease over time. Under this mechanism, the government will allocate initial carbon quotas *G* to the manufacturer based on his previous experience. Then, they trade quotas. Based on the difference between initial carbon quotas and the actual carbon emission, the manufacturer decides to buy or sell quotas in the carbon trading market. After the application of carbon emission reduction technology, the actual carbon emission is $qt(1-e_{i})$, where *t* represents the initial carbon emission per unit product. Thus, the carbon trading amount is

(1) $$E_{i}=qt\left(1-e_{i}\right)-G.$$

Specifically, the manufacturer must purchase an additional carbon quota if his carbon emission exceeds the allocated quota (i.e., $E_{i}>0$), otherwise the surplus can be sold. This form of carbon trading is frequently employed in cap-and-trade mechanism research [2,50,51]. Moreover, we assume that the unit carbon trading price $p_{c}$ is exogenously determined by the carbon trading market. Thus, through the above two steps, the cap-and-trade mechanism can encourage the manufacturer to reduce emissions in a market-oriented way.

The low-carbon product price is linear with the carbon emission reduction and quantity. Consumers are environmentally conscious and ready to pay more for low-carbon products. Based on the emission reduction and green supply chain literature [52,53,54], we assume the inverse demand function is as follows:

(2) $$p_{i}=a-q+be_{i},$$

where *a* is the potential market size and *b* represents the carbon emission sensitivity, which is regarded as consumer environmental awareness. When the consumer environmental awareness and emission reduction rate are improved, the product price will increase.

In order to analyze the effect of the cap-and-trade mechanism and asymmetric information, we consider the following three scenarios. (1) Original scenario, in which the cap-and-trade mechanism is not implemented, and the manufacturer privately has carbon emission reduction information. (2) Implementation scenario, where the cap-and-trade mechanism is put into effect, but information is still asymmetric. (3) Transparency scenario, in which the cap-and-trade mechanism continues to be implemented, but the information becomes transparent. The above three scenarios capture three characteristic stages in the process of policy implementation, which are summarized in Table 2. The implementation scenario is the central case of our research, while the original scenario and transparency scenario are the contrasting cases, without considering asymmetric information or the cap-and-trade mechanism. Moreover, we use the superscript *O*, *I* and *T* to represent the original scenario, implementation scenario and transparency scenario, respectively, in the following analysis.

Moreover, the sequence of events is as follows. Firstly, the manufacturer privately holds the true information of $e_{i}$ and decides a wholesale price *w*. Next, after observing the wholesale price, the retailer infers the type of manufacturer and decides the quantity $q_{i}$. Finally, the market is cleared and the firm’s profits are realized.

## 4. The Equilibrium under Symmetric Information

In this section, emission reduction information $e_{i}$ is common knowledge, providing that the manufacturer discloses emission reduction information to retailer. For example, Baosteel announced that the total carbon emission of a single steel tank is reduced by 15%. This symmetric situation corresponds to the transparency scenario. Then, we discuss optimal decisions and profits of the manufacturer and retailer under the transparency scenario.

To solve the Stackelberg game, we employ backward induction and start with the retailer’s decision. Firstly, given the actual emission reduction rate $e_{i}$ and wholesale price $w_{i}$, the retailer determines her quantity $q_{i}$ as the solution to

(3) $$\max_{q_{i}}\Pi_{ri}^{T}=\left(a-q_{i}+be_{i}-w_{i}\right)q_{i}.$$

Then, the manufacturer decides on the wholesale price $w_{i}$ with the purpose of optimizing his profit

(4) $$\max_{w_{i}}\Pi_{mi}^{T}=w_{i}q_{i}-E_{i}p_{c}.$$

Using the first-order condition, we obtain the equilibrium outcome under this scenario, as shown in Lemma 1.

**Lemma** **1.** *In the transparency scenario, the manufacturer’s optimal wholesale price, the retailer’s optimal product quantity, and their optimal profits are as follows:*

$$\begin{array}{cc} \; & w_{i}^{T*}=\frac{a+be_{i}+p_{c}t\left(1-e_{i}\right)}{2},\\ \; & q_{i}^{T*}=\frac{a+be_{i}-p_{c}t\left(1-e_{i}\right)}{4},\\ \; & \Pi_{mi}^{T*}=\frac{{\left[a+be_{i}-p_{c}t\left(1-e_{i}\right)\right]}^{2}}{8}+Gp_{c},\\ \; & \Pi_{ri}^{T*}=\frac{{\left[a+be_{i}-p_{c}t\left(1-e_{i}\right)\right]}^{2}}{16}. \end{array}$$

## 5. The Equilibrium under Asymmetric Information

In this section, the carbon emission reduction rate $e_{i}$ is the manufacturer’s private information, which is usually not revealed to the retailer. In such a case, there is a dynamic game of incomplete information: the signaling game between the manufacturer and the retailer. The manufacturer is the sender and the retailer is the receiver in the signaling game, where the wholesale price is a message that is a signal for the manufacturer to send emission reduction information to the retailer. Next, we analyze the equilibrium with and without the cap-and-trade mechanism.

### 5.1. Implementation Scenario

In an implementation scenario, the cap-and-trade mechanism is put into effect and the carbon reduction emission information is asymmetric.

Based on the Harsanyi transformation [55,56,57], the timing of this signaling game is as follows. Firstly, “nature” endows the manufacturer with two types of carbon emission reduction rate according to the prior probability distribution; secondly, the manufacturer privately holds the information of $e_{i}$ and decides on wholesale price *w* as a message; thirdly, observing the wholesale price, the retailer updates the beliefs of carbon emission reduction rate, and then determines the order quantity $q_{i}$. Finally, the market is cleared and firms’ profits are realized.

Due to the difference in the signaling strategy, the signaling game will yield multiple equilibria. As is common in the literature, we consider two typical pure strategy perfect Bayesian equilibrium: the separating equilibrium and the pooling equilibrium. The former equilibrium means that different types of the manufacturer determine different wholesale prices; that is, the manufacturer will perfectly reveal the carbon emission reduction rate to the retailer. The latter equilibrium indicates that the manufacturer decides the same wholesale price regardless of his type, so that the retailer cannot infer his type. However, not all equilibria are reasonable. So, we apply the intuitive criterion refinement to refine the unreasonable equilibrium, which is developed by Cho and Kreps [58] and becomes a common refinement principle in the signal game.

The pooling equilibrium is an unreasonable equilibrium, which can be eliminated by the intuitive criterion refinement and a detailed discussion is provided in Appendices Appendix A and Appendix B. This reveals that the pooling equilibrium violates the intuitive criterion. There always exists an intuition that the high-type manufacturer is willing to deviate from the pooling equilibrium by reducing the wholesale price, while the low-type manufacturer is willing to keep the pooling equilibrium. Hence, the pooling equilibrium is an unreasonable equilibrium. Therefore, for the remainder of the paper, we focus on the separating equilibrium uniquely surviving the intuitive criterion refinement.

Under separating equilibrium, relying on her beliefs, the retailer can infer the carbon emission reduction rate from the wholesale price. It is intuitive that the manufacturer, with higher carbon emission reduction which can reduce expenses in the carbon market, will set a lower wholesale price. Thus, the reasonable beliefs regarding the carbon emission reduction rate under separating equilibrium are as follows:

(5) $$\mathrm{Pr}\left(\tilde{e}=e_{h}\right)=\left\{\begin{array}{cc} 1 & if\;w\leq \hat{w},\\ 0 & if\;w>\hat{w}, \end{array}\right.$$

which means that the retailer infers that the manufacturer is high type when the wholesale price is not more than a threshold $\hat{w}$, otherwise they are low type.

Similarly, given a wholesale price $w_{i}$, the retailer’s optimal quantity is given by solving

(6) $$\max_{q_{i}}\Pi_{ri}^{I}=\left(a-q_{i}+be_{i}-w_{i}\right)q_{i},$$

which yields $q_{i}^{*}\left(w_{i}\right)=\frac{a+be_{i}-w_{i}}{2}$. Then, considering the belief structure and response of the retailer, we redefine the following function:

(7) $$V_{ij}\left(w_{j}\right)=\frac{\left(w_{j}-\left(1-e_{i}\right)tp_{c}\right)\left(a+be_{j}-w_{j}\right)}{2}+Gp_{c},\forall i,j\in \left\{h,l\right\}.$$

Here, $V_{ij}\left(w_{i}\right)$ is the manufacturer’s profit if, given the wholesale price $w_{i}$, the retailer deduces the carbon emission reduction rate is $e_{j}$ when the actual carbon emission reduction rate is $e_{i}$. Thus, the manufacturer’s constrained optimization problem is

(8) $$\left\{\begin{array}{c} \max_{w_{h}}V_{hh}\left(w_{h}\right),\\ \max_{w_{l}}V_{ll}\left(w_{l}\right), \end{array}\right.$$

s.t.

(9) $$V_{hh}^{*}\geq V_{hl}\left(w_{l}\right),$$

(10) $$V_{ll}^{*}\geq V_{lh}\left(w_{h}\right),$$

where $V_{hh}^{*}\geq V_{hl}\left(w_{l}\right)$ does not bind, while $V_{ll}^{*}\geq V_{lh}\left(w_{h}\right)$ is binding. This indicates that the low-type manufacturer has an incentive to mimic the high-type one, while the high-type manufacturer has no incentive. As constraint (10) does not always hold, the low-type manufacturer may earn more profit if the low-type manufacturer successfully mimics the high-type. This is because by inducing the retailer to believe that the low-type manufacturer has a high emission reduction, the retailer will increase orders. Thus, the mimicry is favorable to the low-type manufacturer from more orders. On the contrary, for the high-type manufacturer, the potential market size is fixed, and more orders for a low-type manufacturer mean fewer orders for themselves. That is, if the low-type manufacturer succeeds in imitation, the high-type manufacturer’s profits will be hurt. Therefore, the high-type manufacturer is always better off when he delivers information truly, and is unwilling to mimic pricing. Hence, considering only constraint (10), we get the equilibrium solution shown in Lemma 2.

**Lemma** **2.** *In the implementation scenario, the equilibrium outcome and the retailer’s beliefs are as follows:*

*(1) The manufacturer’s optimal wholesale price is*

$$\begin{array}{c} w_{i}^{I*}=\left\{\begin{array}{cc} \frac{a+be_{l}+p_{c}t\left(1-e_{l}\right)}{2}, & if\;\tilde{e}=e_{l};\\ \frac{a+be_{h}+p_{c}t\left(1-e_{l}\right)-M}{2}, & if\;\tilde{e}=e_{h}. \end{array}\right. \end{array}$$

*(2) The retailer’s optimal product quantity is*

$$\begin{array}{c} q_{i}^{I*}=\left\{\begin{array}{cc} \frac{a+be_{l}-p_{c}t\left(1-e_{l}\right)}{4}, & if\;\mathrm{Pr}(\tilde{e}=e_{h})=0;\\ \frac{a+be_{h}-p_{c}t\left(1-e_{l}\right)+M}{4}, & if\;\mathrm{Pr}(\tilde{e}=e_{h})=1, \end{array}\right. \end{array}$$

*consistent with her beliefs that:*

$$\mathrm{Pr}\left(\tilde{e}=e_{h}\right)=\left\{\begin{array}{cc} 1 & if\;w\leq w_{h}^{I*},\\ 0 & if\;w>w_{h}^{I*}. \end{array}\right.$$

*(3) The manufacturer’s optimal profit is*

$$\begin{array}{c} \Pi_{mi}^{I*}=\left\{\begin{array}{cc} \frac{{\left(a+be_{l}-p_{c}t\left(1-e_{l}\right)\right)}^{2}}{8}+Gp_{c}, & if\;\tilde{e}=e_{l};\\ \frac{{\left(a+be_{h}-p_{c}t\left(1-e_{h}\right)\right)}^{2}-{\left(M-p_{c}t\left(e_{h}-e_{l}\right)\right)}^{2}}{8}+Gp_{c}, & if\;\tilde{e}=e_{h}. \end{array}\right. \end{array}$$

*(4) The retailer’s optimal profit is*

$$\begin{array}{c} \Pi_{ri}^{I*}=\left\{\begin{array}{cc} \frac{{\left(a+be_{l}-p_{c}t\left(1-e_{l}\right)\right)}^{2}}{16}, & if\;\mathrm{Pr}(\tilde{e}=e_{h})=0;\\ \frac{{\left(a+be_{h}-p_{c}t\left(1-e_{l}\right)+M\right)}^{2}}{16}, & if\;\mathrm{Pr}(\tilde{e}=e_{h})=1. \end{array}\right. \end{array}$$

*Here, $M\equiv \sqrt{b\left(e_{h}-e_{l}\right)\left(2a+be_{h}+be_{l}-2p_{c}t+2e_{l}p_{c}t\right)}$.*

**Figure 1:**
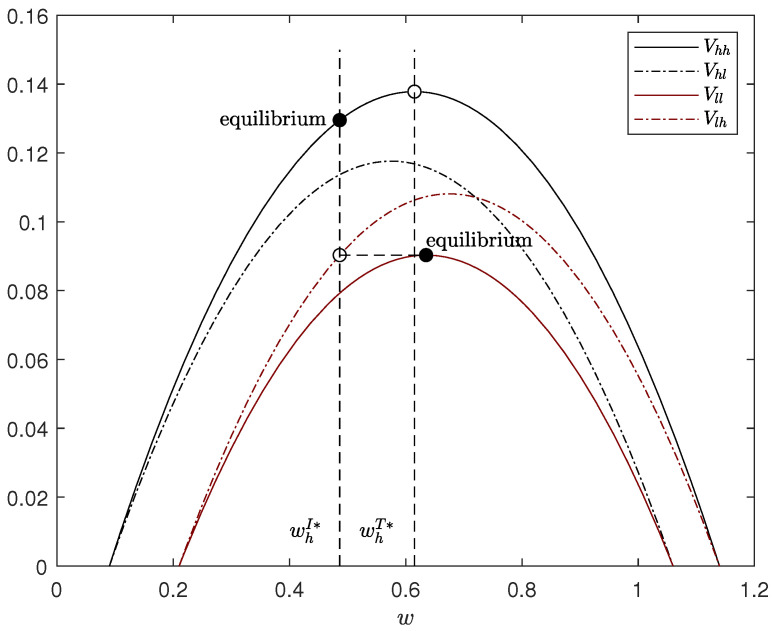
Demonstration of separating equilibrium. The parameters are: t=1, $p_{c}=0.3$, μ=0.5, G=0, a=1, b=0.2, $e_{h}=0.7$ and $e_{l}=0.3$.

According to Lemma 2, the two types of manufacturer have different pricing strategies to separate them successfully, shown in Figure 1, leading to two distinct equilibrium outcomes.

Due to the mimic incentive of the low-type manufacturer, the high-type manufacturer has to distort the wholesale price downward. In detail, if the high-type manufacturer remains having the first-best wholesale price (i.e., $w_{h}^{T*}$), the low-type manufacturer will decide the same price on account of higher mimic profit in the situation where the retailer mistakenly infers the type. For the high-type manufacturer, cutting the wholesale price is the effective means to deal with this mimicry because lower wholesale price reflects higher carbon emission reduction. When the wholesale price drops to $w_{h}^{I*}$ and lower, the low-type manufacturers will suffer more than if they are not mimicking. This price reduction will give rise to profit losses, which means an information cost for the high-type manufacturer accurately passing on his type. Moreover, this information cost will increase with price reduction. Hence, in the pricing range of preventing mimic behavior, that is, $w\leq w_{h}^{I*}$, the optimal wholesale price of the high-type manufacturer is $w_{h}^{I*}$, and can separate the high-type manufacturer from the low-type. However, because no one mimics the low-type manufacturer, his equilibrium outcomes are the same as under complete information, namely the implementation scenario.

### 5.2. Original Scenario

In the original scenario, the carbon reduction emission information is still asymmetric, but the cap-and-trade mechanism is not implemented. Similar to implementation scenario, there is still a signaling game between the manufacturer and the retailer. In this scenario, regarded as a special case of the implementation scenario with G=0 and $p_{c}=0$, only the separating equilibrium is taken into consideration as shown in Lemma 3, while the pooling one is eliminated by the intuitive criterion.

**Lemma** **3.** *In the original scenario, the equilibrium outcomes and the retailer’s beliefs are as follows:*

*(1) The manufacturer’s optimal wholesale price is*

$$\begin{array}{c} w_{i}^{O*}=\left\{\begin{array}{cc} \frac{a+be_{l}}{2}, & if\;\tilde{e}=e_{l};\\ \frac{a+be_{h}-\sqrt{b\left(e_{h}-e_{l}\right)\left(2a+be_{h}+be_{l}\right)}}{2}, & if\;\tilde{e}=e_{h}. \end{array}\right. \end{array}$$

*(2) The retailer’s optimal product quantity is*

$$\begin{array}{c} q_{i}^{O*}=\left\{\begin{array}{cc} \frac{a+be_{l}}{4}, & if\;\mathrm{Pr}(\tilde{e}=e_{h})=0;\\ \frac{a+be_{h}+\sqrt{b\left(e_{h}-e_{l}\right)\left(2a+be_{h}+be_{l}\right)}}{4}, & if\;\mathrm{Pr}(\tilde{e}=e_{h})=1, \end{array}\right. \end{array}$$

*consistent with her beliefs that:*

$$\mathrm{Pr}\left(\tilde{e}=e_{h}\right)=\left\{\begin{array}{cc} 1 & if\;w\leq w_{h}^{O*},\\ 0 & if\;w>w_{h}^{O*}. \end{array}\right.$$

*(3) Regardless of the emission reduction rate, the manufacturer’s optimal profit is*

$$\Pi_{mi}^{O*}=\frac{{\left(a+be_{l}\right)}^{2}}{8}.$$

*(4) The retailer’s optimal profit is*

$$\begin{array}{c} \Pi_{ri}^{O*}=\left\{\begin{array}{cc} \frac{{\left(a+be_{l}\right)}^{2}}{16}, & if\;\mathrm{Pr}(\tilde{e}=e_{h})=0;\\ \frac{{\left(a+be_{h}+\sqrt{b\left(e_{h}-e_{l}\right)\left(2a+be_{h}+be_{l}\right)}\right)}^{2}}{16}, & if\;\mathrm{Pr}(\tilde{e}=e_{h})=1. \end{array}\right. \end{array}$$

From Lemma 3, similarly, only the high-type manufacturer will distort the wholesale price in the original scenario. Whether the cap-and-trade mechanism is implemented or not, there is no change in the incentive to mimic of the two types of manufacturer. In other words, the low-type manufacturer always mimics the high-type to lure the retailer to order more. Hence, the high-type manufacturer decides on a relatively low wholesale price. However, it needs a large degree of distortion to completely separate, which leads the profit of the high-type manufacturer to be the same as the low type’s (i.e., $\Pi_{mh}^{O*}=\Pi_{ml}^{O*}$).

## 6. Analysis

In this section, we first analyze the impact of the cap-and-trade mechanism and asymmetric information on supply chain members by comparing the equilibrium in three scenarios. Then, we consider the influence of the consumer environmental awareness on the equilibrium results.

### 6.1. Impact of Cap-and-Trade Mechanism

In this part, we compare the equilibrium of the manufacturer and the retailer between the implementation scenario and the original scenario to analyze the overall impact of the cap-and-trade mechanism. In addition, we discuss the impact of carbon trading price $p_{c}$ on the equilibrium outcomes.

**Proposition** **1.** *Impact of the cap-and-trade mechanism on manufacturer’s profits is as follows:*

(1) For h-type manufacturer, there exist two thresholds ${\bar{e}}_{h}$ and ${\hat{G}}_{h}=\frac{t}{8}\left[\left(1-e_{l}\right)\left(2a+2be_{l}-p_{c}t+2e_{l}p_{c}t\right)-2\left(e_{h}-e_{l}\right)\left(a+be_{h}-p_{c}t+e_{l}p_{c}t+M\right)\right]$. When $e_{h}>{\bar{e}}_{h}$, $\Pi_{mh}^{I*}>\Pi_{mh}^{O*}$ always holds. When $e_{h}\leq {\bar{e}}_{h}$, $\Pi_{mh}^{I*}>\Pi_{mh}^{O*}$ if and only if $G>{\hat{G}}_{h}$ and vice versa.

(2) For l-type manufacturer, there exists a threshold ${\hat{G}}_{l}=\frac{t}{8}\left(1-e_{l}\right)\left(2a+2be_{l}-p_{c}t+2e_{l}p_{c}t\right)$, such that $\Pi_{ml}^{I*}>\Pi_{ml}^{O*}$ if and only if $G>{\hat{G}}_{l}$ and vice versa.

Proposition 1 investigates how the manufacturer’s profits are affected by the cap-and-trade mechanism. For the high-type manufacturer, when the emission reduction rate is high (i.e., $e_{h}>{\bar{e}}_{h}$), the cap-and-trade mechanism always increases his profits because the carbon emission rate is so high that there can always be superfluous quotas to sell in the carbon trading market. At this time, the manufacturer can always obtain additional benefits in the carbon trading market. However, when the emission reduction rate is low (i.e., $e_{h}\leq {\bar{e}}_{h}$), the intuition that the mechanism has a positive impact only holds when the initial carbon quotas *G* are more than a threshold value ${\hat{G}}_{h}$. That is, the cap-and-trade mechanism makes the manufacturer better off when the received initial carbon quotas are relatively large. If the initial carbon quotas are relatively small, the high-type manufacturer needs to buy quotas in the carbon trading market that will lead to additional costs. Similarly, for the low-type manufacturer, the impact of the cap-and-trade mechanism depends on the initial carbon quotas *G*. When the initial carbon quotas $G>{\hat{G}}_{l}$, the cap-and-trade mechanism is beneficial to the low-type manufacturer. However, when $G\leq {\hat{G}}_{l}$, this mechanism harms the low-type manufacturer. When the carbon quota reaches a certain threshold, it is intuitive that the cap-and-trade mechanism is beneficial to manufacturers, which shows that loose initial carbon quotas can give the manufacturer the opportunity to make profits through carbon trading.

**Figure 2:**
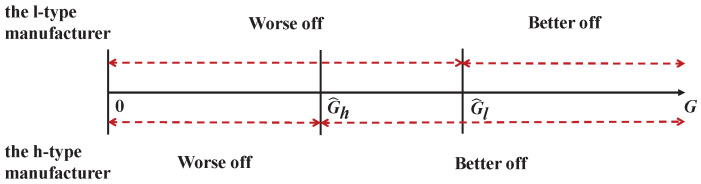
Illustration of Performance Comparison.

We can also find that ${\hat{G}}_{l}>{\hat{G}}_{h}$ for any given parameters when $e_{h}\leq \;{\bar{e}}_{h}$. This shows that when the high-type manufacturer has low emission reduction (i.e., $e_{h}\leq {\bar{e}}_{h}$), reasonable initial carbon quotas $G\in [{\hat{G}}_{h},{\hat{G}}_{l}]$ can effectively inspire emission reduction, as shown in Figure 2.

If the initial carbon quotas are relatively low (i.e., $G<{\hat{G}}_{h}$), it is adverse for both types of the manufacturer that the carbon quotas are insufficient and need to be purchased. If the initial carbon quotas are relatively high (i.e., $G>{\hat{G}}_{h}$), the cap-and-trade mechanism is good for the manufacturer regardless of emission reduction rate, but it is difficult to control carbon emissions. Only when the quota is within a reasonable range can the cap-and-trade mechanism benefit the high-type manufacturer, but not the low-type manufacturer. Consequently, the low-type manufacturer is encouraged to improve the emission reduction rate and strives to improve the level of emission reduction to turn himself into the high-type.

**Figure 3:**
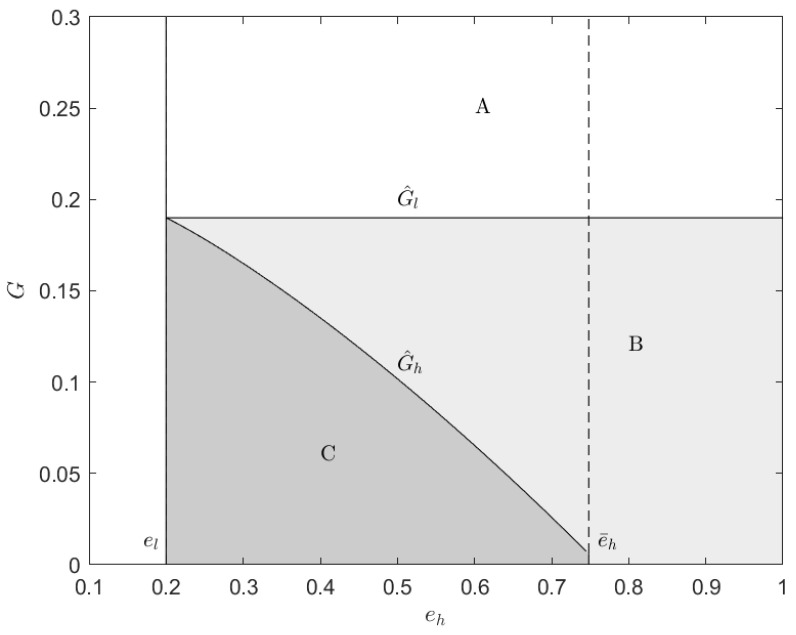
Illustration of Different Impacts of the cap-and-trade Mechanism. The parameters are t=1, $p_{c}=0.3$, μ=0.5, G=0, a=1, b=0.2, and $e_{l}=0.2$.

Furthermore, Figure 3 shows the different impacts of the cap-and-trade mechanism on the manufacturer in different parameter areas. According to the different impacts, this figure is divided into three areas. In area A, reflecting $G>{\hat{G}}_{l}$, the cap-and-trade mechanism makes both types of manufacturer better off. In area B, only the high-type manufacturer is better off. In other words, as stated earlier, region B is the feasible area for the initial carbon quotas. In area C, both types of manufacturer are worse off. Thus, this figure vividly integrates Proposition 1 and the relationship between ${\hat{G}}_{h}$ and ${\hat{G}}_{l}$. Only under certain conditions can this mechanism effectively motivate the high-type manufacturer. Before formulating the cap-and-trade mechanism, it is necessary for the government to collect as much information as possible about carbon emission reduction of manufacturers. After this step, they need to analyze the information in depth and try to determine the initial carbon quota based on our findings. Otherwise, the manufacturer with a high emission reduction level will suffer from a loss of profits after implementing the policy, which will reduce their emission reduction motivation.

**Proposition** **2.** No matter what type of manufacturer, $w_{i}^{I*}>w_{i}^{O*}$, $q_{i}^{I*}<q_{i}^{O*}$ and $\Pi_{ri}^{I*}<\Pi_{ri}^{O*}$ always hold.

Proposition 2 indicates that the type of manufacturer will not change the impact of the cap-and-trade mechanism on the wholesale price, quantity, and retailer’s profit. Under the cap-and-trade mechanism, the optimal wholesale price is unrelated to the initial quotas *G*. Because, when the manufacturer decides the wholesale price, he always considers the possibility of paying costs in the carbon trading market as long as he has carbon emissions. So, no matter whether the emission reduction rate is high or low, the manufacturer will raise the wholesale price. In other words, the cap-and-trade mechanism incurs an increase in the wholesale price. Naturally, the quantity is lower under this mechanism. Furthermore, with the reduction of orders and price markup (i.e., $p_{i}-w_{i}$), the cap-and-trade mechanism reduces the profit of the retailer.

**Corollary** **1.** 1. $\frac{\partial w_{i}^{T/I*}}{\partial p_{c}}>0$, $\frac{\partial q_{i}^{T/I*}}{\partial p_{c}}<0$, $\frac{\partial \Pi_{ri}^{T/I*}}{\partial p_{c}}<0$.

*2. There exist three thresholds $G_{1}=\frac{t\left(1-e_{h}\right)\left(a+be_{h}-\left(1-e_{h}\right)p_{c}t\right)}{4}$, $G_{2}=\frac{t\left(1-e_{l}\right)\left(a+be_{l}-\left(1-e_{l}\right)p_{c}t\right)}{4}$ and $G_{3}=\frac{t(\left(1-e_{h}\right)a+\left(e_{l}-e_{h}^{2}-e_{l}^{2}+2e_{l}\right)b-\left(1-e_{l}\right)\left(1+e_{l}-2e_{h}\right)p_{c}t)}{4}-\frac{bt(2a+\left(e_{h}+e_{l}\right)b-3\left(1-e_{l}\right)p_{c}t)}{4M}$.*

When $G<G_{1}$, $\frac{\partial \Pi_{mh}^{T*}}{\partial p_{c}}<0$; when $G>G_{1}$, $\frac{\partial \Pi_{mh}^{T*}}{\partial p_{c}}>0$.

When $G<G_{2}$, $\frac{\partial \Pi_{ml}^{T/I*}}{\partial p_{c}}<0$; when $G>G_{2}$, $\frac{\partial \Pi_{ml}^{T/I*}}{\partial p_{c}}>0$.

When $G<G_{3}$, $\frac{\partial \Pi_{mh}^{I*}}{\partial p_{c}}<0$; when $G>G_{3}$, $\frac{\partial \Pi_{mh}^{I*}}{\partial p_{c}}>0$.

Corollary 1 shows the changing trends in equilibrium outcomes, with respect to carbon trading price. With the rise of carbon trading price, the optimal wholesale price will rise while the optimal order quantity and optimal retailer profits will decline. As the higher carbon trading price means that manufacturer’s transaction costs in the carbon trading market will increase, the manufacturer will raise wholesale prices. Naturally, the quantity is lower which leads to lower retailer profits. Meanwhile, this corollary is also the confirmation of Proposition 2. It is easy to spot the fact that the optimal wholesale price, order quantity, and retailer’s profit are affected by the carbon trading price, but not the initial carbon quotas. In this case, discussing the carbon trading price can fully represent the impact of the cap-and-trade mechanism. Consequently, Proposition 2 is equivalent to investigate the impact of carbon trading price increase from 0 in an implementation scenario. Moreover, as for the manufacturer, whether the profit increases or decreases with carbon trading price depends on the initial quotas. In general, when the initial quotas are relatively low, the manufacturer’s profit decreases in the carbon trading price; whereas, when the initial quotas are relatively high, the manufacturer’s profit increases with the carbon trading price. However, the threshold of the initial quota in different scenarios is different. In contrast to Xu et al. [26], who believe that both the optimal profit of the manufacturer and the retailer decrease with the increase in carbon trading price, our analysis shows that carbon trading price and the initial quota will jointly affect the profits of supply chain members. Specifically, when the initial quota is large, the manufacturer’s optimal profit increases with the carbon trading price, and vice versa. However, the retailer’s profit always decreases with the carbon trading price. Our results for the effect of the cap-and-trade mechanism on the profits of supply chain members are consistent with some empirical observations.

### 6.2. Impact of Asymmetric Information

In this part, we compare the equilibrium of the manufacturer and the retailer between the implementation scenario and transparency scenario to analyze the impact of asymmetric information. Intuitively, the manufacturer with sufficient information is often in a favorable position, and can obtain remuneration beyond the value of goods by virtue of information advantages. However, this is not always true for different types of the manufacturer.

**Proposition** **3.** For h-type manufacturer, $\Pi_{mh}^{I*}<\Pi_{mh}^{T*}$ always holds. For l-type manufacturer, $\Pi_{ml}^{I*}=\Pi_{ml}^{T*}$ always holds.

Proposition 3 reveals that asymmetric information would decrease the high-type manufacturer’s total (two-stage) profit, while increasing the low-type manufacturer’s total (two-stage) profit. Asymmetric information will give rise to the signaling effect. That is, as is stated above, the high-type manufacturer has to distort his wholesale price to truly reveal his high emission reduction rate while the low-type manufacturer can maintain the first-best wholesale price. This generates the information cost only for the high-type manufacturer which reduces his profits. However, the low-type manufacturers can make the same profit regardless of whether the information is symmetrical or not. Thus, compared with the low-type manufacturer, the high-type manufacturer prefers information symmetry. In contrast to Li et al. [44], who believe that H-type manufacturers can only obtain reserved income, while L-type manufacturers can obtain additional income, our analysis shows that the manufacturer with private information is in a disadvantageous position. It is difficult to obtain additional profits, or even the profits of the manufacturer with high emission reduction level will be damaged. Therefore, it is necessary to eliminate the signaling effect caused by asymmetric information, so as to prevent the high-type manufacturer from a lack of emission reduction power by maintaining first-best profits. Our results for the impact of asymmetric information on a firm are consistent with some empirical observations that the government needs to strengthen supervision and improve the transparency of environmental information to encourage enterprises to innovate and reduce emissions [59].

In addition, the manufacturer with a high emission reduction rate, generally speaking, can obtain more benefits from the carbon trading market and bargaining with the retailer. In other words, the high-type manufacturer has the emission reduction advantage that can be described as $\Delta =\Pi_{mh}^{*}-\Pi_{ml}^{*}$. Then, we get the following proposition to investigate the effect of asymmetric information on emission reduction advantage.

**Proposition** **4.** *Comparing the emission reduction advantages of the three scenarios, we get $\Delta^{T}>\Delta^{I}>\Delta^{O}=0$.*

Proposition 4 indicates that asymmetric information weakens emission reduction advantage. Because of the signaling effect, the high-type manufacturer gains less profit than that under complete information, that is, less emission reduction advantage. Therefore, under complete information, that is, the transparency scenario, the high type manufacturer can obtain the greatest emission reduction advantage. However, this negative effect of asymmetric information is different in the implementation scenario and original scenario. In the original scenario, the high-type manufacturer distorts the wholesale price to a great extent, which makes him lose advantage completely, that is, $\Delta^{O}=0$. In this case, the emission reduction advantage is quite small, which can be easily eliminated by asymmetric information. However, in the implementation scenario, owing to the cap-and-trade mechanism, asymmetric information just weakens the partial advantage. It further illustrates that the cap-and-trade mechanism is favorable for the high-type manufacturer by amplifying the emission reduction advantage.

**Proposition** **5.** If the manufacturer is high type, $q_{h}^{T*}<q_{h}^{I*}$ and $\Pi_{rh}^{T*}<\Pi_{rh}^{I*}$. If the manufacturer is low type, $q_{l}^{T*}=q_{l}^{I*}$ and $\Pi_{rl}^{T*}=\Pi_{rl}^{I*}$.

Proposition 5 shows how asymmetric information affects the retailer, including her order quantity and profit. If the manufacturer has a high emission reduction rate, asymmetric information will increase the order quantity and the retailer’s profit. As mentioned above, the manufacturer needs to reduce the wholesale price to convey his type under asymmetric information. Thus, the retailer will adjust the order quantity to maximize her profit. If the manufacturer has a low emission reduction rate, asymmetric information will not have an impact on retailers. In this case, the equilibrium is consistent with that in complete information.

### 6.3. Impact of Consumer Environmental Awareness

In this part, we discuss the impact of consumer environmental awareness *b* on the equilibrium outcomes under the three scenarios.

**Corollary** **2.** 1. $\frac{\partial q^{*}}{\partial b}>0$ in all scenarios.

2. (1) If the manufacturer is a low type, $\frac{\partial w_{l}^{*}}{\partial b}>0$ in all scenarios.

(2) If the manufacturer is a high type, $\frac{\partial w_{h}^{T*}}{\partial b}>0$. When $b<\frac{\left(e_{h}-e_{l}\right)\left(a-p_{c}t\left(1-e_{l}\right)\right)}{e_{l}\left(e_{h}+e_{l}\right)}$, $\frac{\partial w_{h}^{I*}}{\partial b}<0$; when $b>\frac{\left(e_{h}-e_{l}\right)\left(a-p_{c}t\left(1-e_{l}\right)\right)}{e_{l}\left(e_{h}+e_{l}\right)}$, $\frac{\partial w_{h}^{I*}}{\partial b}>0$. When $b<\frac{a(e_{h}-e_{l})}{e_{l}\left(e_{h}+e_{l}\right)}$, $\frac{\partial w_{h}^{O*}}{\partial b}<0$; when $b>\frac{a(e_{h}-e_{l})}{e_{l}\left(e_{h}+e_{l}\right)}$, $\frac{\partial w_{h}^{O*}}{\partial b}>0$.

3. $\frac{\partial \Pi_{m}^{*}}{\partial b}>0$, $\frac{\partial \Pi_{r}^{*}}{\partial b}>0$ in all scenarios.

Corollary 2 indicates that the effect of consumer environmental awareness on the optimal quantity, manufacturer’s profit, and retailer’s profit is the same in each scenario, but the impact on the optimal wholesale price is different. Firstly, the optimal quantity increases with the consumer environmental awareness in all scenarios. As the higher consumer environmental awareness means that consumers are willing to pay higher prices, the retailer will order more from the manufacturer (i.e., $\frac{\partial q^{*}}{\partial b}>0$). This shows that the consumer environmental awareness is instrumental in opening the product market, which will not be affected by asymmetric information and the cap-and-trade mechanism.

**Figure 4:**
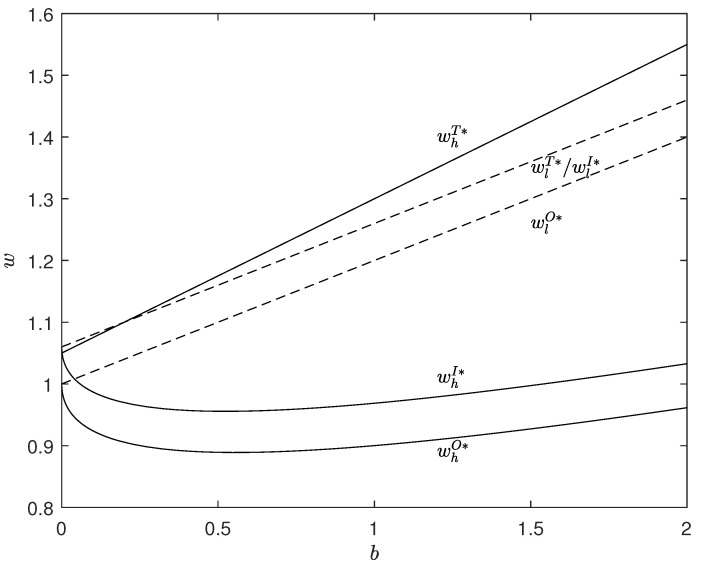
Illustration of Different Impacts of the Consumer Environmental Awareness on Optimal Wholesale Price. The parameters are: t=1, $p_{c}=0.3$, μ=0.5, G=0, a=2, $e_{h}=0.5$ and $e_{l}=0.4$.

Secondly, for the optimal wholesale price, the impact of consumer environmental awareness is different for different types of the manufacturer, as shown in Figure 4. This is because consumer environmental awareness has two opposite effects on the wholesale price. One is a positive effect that is to increase the optimal wholesale price by increasing the optimal order quantity, and the other is a negative effect that is to reduce the optimal wholesale price by increasing the degree of distortion. For the low-type manufacturer without distortion, the consumer environmental awareness only plays a positive role. Hence, if the manufacturer has a low carbon emission reduction rate, the optimal wholesale price will always increase with the improvement in consumer environmental awareness. Conversely, for the high-type manufacturer, the two effects work together on the optimal wholesale price under asymmetric information, while the positive effect works under symmetric information. Under asymmetric information, when the consumer environmental awareness is relatively low, the negative effect is dominant rather than the positive effect; whereas, when the consumer environmental awareness is relatively high, the positive effect occupies a dominant position. Such effects are consistent, and any increase in consumer environmental awareness eventually makes the wholesale price increase first and decrease later. Under symmetric information, similar to the low-type manufacturer, there is no distortion. So, the optimal wholesale price is positively correlated with the consumer environmental awareness in the transparency scenario.

Thirdly, the improvement of consumer environmental awareness results in the increase in the optimal profits of the manufacturer and the retailer. The manufacturer’s profit is positively correlated with order quantity and unit net profit. When the consumer environmental awareness increases, the optimal quantity and unit net profit increase, and so does the optimal profit of the manufacturer. Moreover, the retailer’s profit is closely related to the order quantity. As is stated above, there is always an intuition that the retailer’s profit will increase with the order quantity. So, the rise in the retailer’s profit is paralleled by an increase in the consumer environmental awareness. This indicates that both the manufacturer and the retailer prefer consumers with high environmental awareness, which can improve their performance.

## 7. Conclusions

In this paper, we consider a supply chain under the cap-and-trade mechanism, which consists of one manufacturer who has private control over the emission reduction rate, and one retailer. We investigate three scenarios to explore the impact of the cap-and-trade mechanism and asymmetric information. The main conclusions are as follows.

- *How will the manufacturer and retailer determine optimal decisions to maximize their own profits?* Using the game model, we get the equilibrium results under each scenario. Under information symmetry, both the manufacturer and the retailer can obtain the first-best profits. Under the condition of asymmetric information, the situation becomes different, that is, the high-type manufacturer will distort the wholesale price, which will cause changes in the optimal decisions of supply chain members.
- *What is the impact of the cap-and-trade mechanism on the performances of the supply chain members?* The cap-and-trade mechanism can effectively encourage the manufacturer to reduce emissions only when the initial quota is in a relatively middle area, in which the high-type manufacturer is better off while the low-type manufacturer is worse off. In addition, information asymmetry also weakens the emission reduction advantages of the high-type manufacturer. The initial quotas will determine whether the manufacturer’s profit increases or decreases with the carbon trading price. Moreover, the cap-and-trade mechanism will increase wholesale prices, but reduce quantity. With the reduction in orders and price markup, the profit of the retailer is reducing.
- *What effect does asymmetric information about carbon emission reduction have on the performance of supply chain members?* It is not always beneficial for manufacturers to have private information on emission reduction rate. Under information asymmetry, the high-type manufacturer will distort the wholesale price, which results in the loss of profits. According to different types of manufacturer, the impact of information asymmetry on retailers’ orders and profits is also different. However, the retailer cannot perform better.

Moreover, this paper provides the following managerial implications for the cap-and-trade mechanism development under asymmetric information. On the one hand, with regard to the initial carbon quota allocation, we find a reasonable range from the perspective of the manufacturer’s own current emission reduction technology. The present distribution system is mainly formulated from historical emissions (grandfathering method) and industry conditions (benchmarking method). The total amount and distribution of quotas are still set in advance based on historical data. However, due to the uncertainty of emission reduction technology innovation, the initial carbon quotas may deviate from the reality. So, based on the fact that the manufacturer’s own technology is often uncertain and asymmetric, we find that only a reliable initial carbon quota range can effectively encourage manufacturers to reduce emissions. This provides a new perspective for the determination of initial carbon quotas. From the perspective of carbon policy makers, collecting detailed information and determining the initial carbon quota level are two important tasks that must be completed before designing this policy. On the other hand, with regard to carbon emission disclosure, manufacturers with higher carbon emissions should actively disclose more detailed carbon emission information, and the government should improve the disclosure standards. For example, digital technology is an effective way to realize online emission reduction interconnections such as Internet of Things, cloud computing, and big data.

There are many aspects that can be expanded on in the cap-and-trade mechanism problem under the asymmetric information of emission reduction. Firstly, we assume that the manufacturer sells products through only one retailer. In fact, the manufacturer may sell through multiple distribution channels. If we consider the competition of downstream enterprises, more interesting conclusions may be drawn. Secondly, we only consider that there is only one product in the supply chain. However, the manufacturer often carries out diversified production, and it will be meaningful to consider multi-products. Thirdly, we consider a single period problem. Actually, product production, sales, and the cap-and-trade mechanism are a continuous and lasting process. If we consider a multi-period problem, more valuable conclusions may appear. For example, carbon quotas from the current period can be traded in the next period.

## Figures and Tables

**Table 1:** Key similarities and diferences in literature.

Literature	Asymmetric Information	Cap-and-Trade Mechanism	Consumer Environmental Awareness	Supply Chain
[43]	carbon reduction efficiency			
[44]	carbon reduction efficiency			✓
[45]	carbon reduction efficiency		✓	
[46]	carbon reduction efficiency		✓	✓
[47]	carbon reduction efficiency		✓	✓
[48]	carbon reduction efficiency	✓		✓
[49]	carbon reduction efficiency	✓		✓
[17]	low-carbon preference	✓	✓	
this paper	carbon reduction efficiency	✓	✓	✓

**Table 2:** The three scenarios.

	Asymmetry	Symmetry
Cap-and-trade	implementation scenario	transparency scenario
No Cap-and-trade	original scenario	/

**Figure A1:**
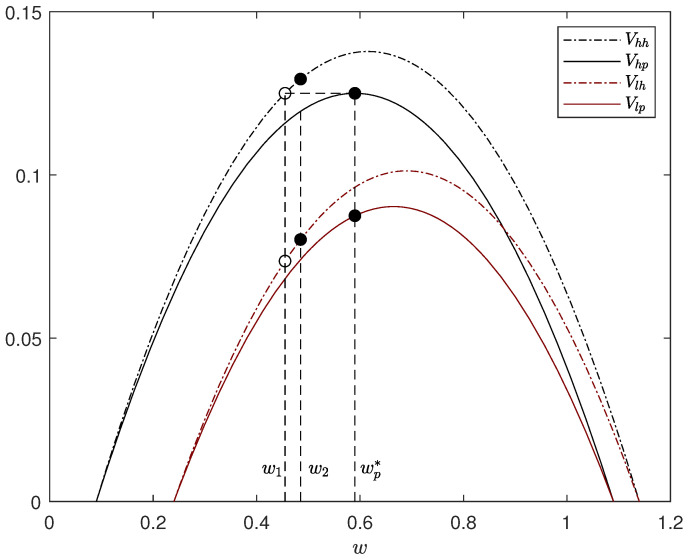
Demonstration of the refinement over pooling equilibria by the intuitive criterion. The parameters are: t=1, $p_{c}=0.3$, μ=0.5, G=0, a=1, b=0.2, $e_{h}=0.7$ and $e_{l}=0.2$.

## Data Availability

Not applicable.

## Appendix A. Proofs

**Proof of Lemma** **1.** Given the wholesale price $w_{i}$, the retailer’s optimal quantity can be obtained to maximize its profit:

$$q_{i}^{*}\left(w_{i}\right)=\arg\max_{q_{i}}\left(a-q_{i}+be_{i}-w_{i}\right)q_{i}=\frac{a+be_{i}-w_{i}}{2}.$$

Similarly, the manufacturer’s optimal wholesale price can be solved:

$$\begin{array}{cc} w_{i}^{*} & =\arg\max_{w_{i}}\left(w-\left(1-e_{i}\right)tp_{c}\right)q_{i}^{*}\left(w_{i}\right)+Gp_{c}\\ \; & =\frac{a+be_{i}+p_{c}t\left(1-e_{i}\right)}{2}. \end{array}$$

Bring this back to the equation of $q_{i}^{*}\left(w_{i}\right)$ and profit functions, we can derive the optimums. □

**Proof of Lemma** **2.** After simplification, the above manufacturer’s optimization problem is

(A1) $$\left\{\begin{array}{c} \max_{w_{h}}\frac{\left(w_{h}-\left(1-e_{h}\right)tp_{c}\right)\left(a+be_{h}-w_{h}\right)}{2}+Gp_{c},\\ \max_{w_{l}}\frac{\left(w_{l}-\left(1-e_{l}\right)tp_{c}\right)\left(a+be_{l}-w_{l}\right)}{2}+Gp_{c}, \end{array}\right.$$

s.t.

(A2)
$${\left[a+be_{h}-p_{c}t\left(1-e_{h}\right)\right]}^{2}\geq 4\left(w_{l}-\left(1-e_{h}\right)tp_{c}\right)\left(a+be_{l}-w_{l}\right),$$

(A3)
$${\left[a+be_{l}-p_{c}t\left(1-e_{l}\right)\right]}^{2}\geq 4\left(w_{h}-\left(1-e_{l}\right)tp_{c}\right)\left(a+be_{h}-w_{h}\right),$$

Firstly, constrain (A2) does not bind, showing that high-type manufacturers are not willing to mimic the pricing behavior of the low-type. So, it is unnecessary for low-type manufacturers to consider the mimicry when deciding on the wholesale price. Additionally, the equilibrium outcome of a low-type manufacturer is the same as that in the transparency scenario. Second, due to binding constrain (A3), the mimic incentive of low-type manufacturers should be considered in the optimization problem of the high-type manufacturer:

$$\begin{array}{cc} \; & \max_{w_{h}}\frac{\left(w_{h}-\left(1-e_{h}\right)tp_{c}\right)\left(a+be_{h}-w_{h}\right)}{2}+Gp_{c},\\ \; & s.t.\\ \; & {\left[a+be_{l}-p_{c}t\left(1-e_{l}\right)\right]}^{2}\geq 4\left(w_{h}-\left(1-e_{l}\right)tp_{c}\right)\left(a+be_{h}-w_{h}\right). \end{array}$$

According to the constraint, we can get $w_{h}\leq {\underline{w}}_{h}$ or $w_{h}\geq {\bar{w}}_{h}$, where ${\underline{w}}_{h}=$$\frac{a+be_{h}+p_{c}t-e_{l}p_{c}t-\sqrt{b\left(e_{h}-e_{l}\right)\left(2a+be_{h}+be_{l}-2p_{c}t+2e_{l}p_{c}t\right)}}{2}$ and ${\bar{w}}_{h}=$$\frac{a+be_{h}+p_{c}t-e_{l}p_{c}t+\sqrt{b\left(e_{h}-e_{l}\right)\left(2a+be_{h}+be_{l}-2p_{c}t+2e_{l}p_{c}t\right)}}{2}$. Moreover, $V_{hh}\left(w_{h}\right)$ is a quadratic function with respect to $w_{h}$, which monotonically increases with $w_{h}$ when $w_{h}<\frac{a+be_{h}+p_{c}t\left(1-e_{h}\right)}{2}\equiv w_{h^{\prime }}$ and decreases when $w_{h}>w_{h^{\prime }}$. As ${\underline{w}}_{h}<w_{h^{\prime }}<{\bar{w}}_{h}$ and $w_{h^{\prime }}-{\underline{w}}_{h}<{\bar{w}}_{h}-w_{h^{\prime }}$ are always true, we can obtain that the equilibrium wholesale price is $w_{h}^{I*}={\underline{w}}_{h}$. Making further efforts, we can get the equilibrium solution. □

**Proof of Proposition** **1.** First, comparing the high-type manufacturer’s profit in an implementation scenario with that in original scenario, we have

$$\begin{array}{cc} \Pi_{mh}^{I*}-\Pi_{mh}^{O*}= & \frac{\left(a+be_{h}-p_{c}t\left(1-e_{l}\right)+M\right)\left(a+be_{h}-\left(1+e_{l}-2e_{h}\right)p_{c}t-M\right)}{8}\\ & +Gp_{c}-\frac{{\left(a+be_{l}\right)}^{2}}{8}, \end{array}$$

which is a monotone function of G. Hence, it has one and only one root

$$\begin{array}{cc} {\hat{G}}_{h} & =\frac{{\left(a+be_{l}\right)}^{2}-\left(a+be_{h}-p_{c}t\left(1-e_{l}\right)+M\right)\left(a+be_{h}-\left(1+e_{l}-2e_{h}\right)p_{c}t-M\right)}{8p_{c}}\\ \; & =\frac{t}{8}\left[\left(1-e_{l}\right)\left(2a+2be_{l}-p_{c}t+2e_{l}p_{c}t\right)-2\left(e_{h}-e_{l}\right)\left(a+be_{h}-p_{c}t+e_{l}p_{c}t+M\right)\right]. \end{array}$$

When $e_{h}\geq {\bar{e}}_{h}=\frac{\left(a-p_{c}t-be_{l}+2be_{l}^{2}+e_{l}^{2}p_{c}t\right)K-\sqrt{b{\left(1-e_{l}\right)}^{3}K^{3}}+e_{l}p_{c}t\left(1-e_{l}\right)\left(a+be_{l}\right)}{2a^{2}+2bae_{l}-bp_{c}te_{l}^{2}+bp_{c}te_{l}-\left(2b+2p_{c}t-3be_{l}-2e_{l}p_{c}t\right)K}$, where $K=2a+2be_{l}-p_{c}t+e_{l}p_{c}t$, ${\hat{G}}_{h}<0$. In this case, $\Pi_{mh}^{I*}-\Pi_{mh}^{O*}>0$. When $e_{h}<{\bar{e}}_{h}$, ${\hat{G}}_{h}>0$. In this case, when $e_{h}\leq {\bar{e}}_{h}$, $\Pi_{mh}^{I*}>\Pi_{mh}^{O*}$ if and only if $G>{\hat{G}}_{h}$ and vice versa. Then, comparing the low-type manufacturer’s profit in the implementation scenario with that in original scenario, we have

$$\Pi_{ml}^{I*}-\Pi_{ml}^{O*}=\frac{{\left(a+be_{l}-p_{c}t\left(1-e_{l}\right)\right)}^{2}}{8}+Gp_{c}-\frac{{\left(a+be_{l}\right)}^{2}}{8}.$$

Similarly, it has one and only one root

$${\hat{G}}_{l}=\frac{t}{8}\left(1-e_{l}\right)\left(2a+2be_{l}-p_{c}t+2e_{l}p_{c}t\right)>0.$$

So, $\Pi_{ml}^{I*}>\Pi_{ml}^{O*}$ if and only if $G>{\hat{G}}_{l}$, and vice versa. Last, comparing the two thresholds ${\hat{G}}_{h}$ and ${\hat{G}}_{l}$ when $e_{h}\leq {\bar{e}}_{h}$, we have

$${\hat{G}}_{h}-{\hat{G}}_{l}=-\frac{t}{4}\left(e_{h}-e_{l}\right)\left(a+be_{h}-p_{c}t+e_{l}p_{c}t+M\right),$$

which is negative. □

**Proof of Proposition** **2.** First, we consider the low-type manufacturer. Comparing the wholesale price and quantity in the implementation scenario with that in original scenario, we have

$$w_{l}^{I*}-w_{l}^{O*}=\frac{a+be_{l}+p_{c}t\left(1-e_{l}\right)}{2}-\frac{a+be_{l}}{2}=\frac{p_{c}t\left(1-e_{l}\right)}{2}>0$$

$$q_{l}^{I*}-q_{l}^{O*}=\frac{a+be_{l}-p_{c}t\left(1-e_{l}\right)}{4}-\frac{a+be_{l}}{4}=-\frac{p_{c}t\left(1-e_{l}\right)}{4}<0$$

Then, we consider the high-type manufacturer. Comparing the wholesale price and quantity in the implementation scenario with that in the original scenario, we have

$$\begin{array}{cc} w_{h}^{I*} & -w_{h}^{O*}=\frac{a+be_{h}+p_{c}t\left(1-e_{l}\right)-M}{2}-\frac{a+be_{h}-\sqrt{b\left(e_{h}-e_{l}\right)\left(2a+be_{h}+be_{l}\right)}}{2}\\ \; & =\frac{p_{c}t\left(1-e_{l}\right)+\sqrt{b(e_{h}-e_{l})}\left(\sqrt{2a+be_{h}+be_{l}}-\sqrt{2a+be_{h}+be_{l}-2p_{c}t\left(1-e_{l}\right)}\right)}{2}>0. \end{array}$$

$$\begin{array}{cc} q_{h}^{I*} & -q_{h}^{O*}=\frac{a+be_{h}-p_{c}t\left(1-e_{l}\right)+M}{4}-\frac{a+be_{h}+\sqrt{b\left(e_{h}-e_{l}\right)\left(2a+be_{h}+be_{l}\right)}}{4}\\ \; & =-\frac{p_{c}t\left(1-e_{l}\right)+\sqrt{b(e_{h}-e_{l})}\left(\sqrt{2a+be_{h}+be_{l}}-\sqrt{2a+be_{h}+be_{l}-2p_{c}t\left(1-e_{l}\right)}\right)}{4}<0 \end{array}$$

Last, we take the retailer into consideration.

$$\begin{array}{c} \Pi_{ri}^{I*}-\Pi_{ri}^{O*}={\left(q_{i}^{I*}\right)}^{2}-{\left(q_{i}^{O*}\right)}^{2}=\left(q_{h}^{I*}+q_{h}^{O*}\right)\left(q_{h}^{I*}-q_{h}^{O*}\right)<0 \end{array}$$

□

**Proof of Corollary** **1.** First, we find the derivative of the optimal wholesale price, with respect to the carbon trading price under various scenarios.

$$\begin{array}{cc} \; & \frac{\partial w_{i}^{T*}}{\partial p_{c}}=\frac{(1-e_{i})t}{2}>0,\\ \; & \frac{\partial w_{l}^{I*}}{\partial p_{c}}=\frac{(1-e_{l})t}{2}>0,\\ \; & \frac{\partial w_{h}^{I*}}{\partial p_{c}}=\frac{(1-e_{h})t}{2}+\frac{\left(e_{h}-e_{l}\right)\left(1-e_{l}\right)bt}{2M}>0. \end{array}$$

Then, we find the derivative of the optimal quantity with respect to the carbon trading price under various scenarios.

$$\begin{array}{cc} \; & \frac{\partial q_{i}^{T*}}{\partial p_{c}}=-\frac{(1-e_{i})t}{4}<0\\ \; & \frac{\partial q_{l}^{I*}}{\partial p_{c}}=-\frac{(1-e_{i})t}{4}<0\\ \; & \frac{\partial q_{h}^{I*}}{\partial p_{c}}=-\frac{(1-e_{i})t}{4}-\frac{\left(e_{h}-e_{l}\right)\left(1-e_{l}\right)bt}{4M}<0 \end{array}$$

Moreover, we can get

$$\frac{\partial \Pi_{ri}^{*}}{\partial p_{c}}=\frac{\partial {\left(q_{i}^{*}\right)}^{2}}{\partial p_{c}}=2q_{i}^{*}\frac{\partial q_{i}^{*}}{\partial p_{c}}<0$$

Finally, we find the derivative of the optimal manufacturer profit with respect to the carbon trading price under various scenarios.

$$\begin{array}{cc} \frac{\partial \Pi_{mh}^{T*}}{\partial p_{c}}= & G-\frac{t\left(1-e_{h}\right)\left(a+be_{h}-\left(1-e_{h}\right)p_{c}t\right)}{4}\\ \frac{\partial \Pi_{ml}^{T*}}{\partial p_{c}}= & \frac{\partial \Pi_{ml}^{I*}}{\partial p_{c}}=G-\frac{t\left(1-e_{l}\right)\left(a+be_{l}-\left(1-e_{l}\right)p_{c}t\right)}{4}\\ \frac{\partial \Pi_{mh}^{I*}}{\partial p_{c}}= & G-\frac{t(\left(1-e_{h}\right)a+\left(e_{l}-e_{h}^{2}-e_{l}^{2}+2e_{l}\right)b-\left(1-e_{l}\right)\left(1+e_{l}-2e_{h}\right)p_{c}t)}{4}\\ & -\frac{bt(2a+\left(e_{h}+e_{l}\right)b-3\left(1-e_{l}\right)p_{c}t)}{4M} \end{array}$$

Let $\frac{\partial \Pi_{mh}^{T*}}{\partial p_{c}}=0$, $\frac{\partial \Pi_{ml}^{T*}}{\partial p_{c}}=\frac{\partial \Pi_{ml}^{I*}}{\partial p_{c}}=0$ and $\frac{\partial \Pi_{mh}^{I*}}{\partial p_{c}}=0$, we can get $G_{1}=\frac{t\left(1-e_{h}\right)\left(a+be_{h}-\left(1-e_{h}\right)p_{c}t\right)}{4}$, $G_{2}=\frac{t\left(1-e_{l}\right)\left(a+be_{l}-\left(1-e_{l}\right)p_{c}t\right)}{4}$ and $G_{3}=\frac{t(\left(1-e_{h}\right)a+\left(e_{l}-e_{h}^{2}-e_{l}^{2}+2e_{l}\right)b-\left(1-e_{l}\right)\left(1+e_{l}-2e_{h}\right)p_{c}t)}{4}-\frac{bt(2a+\left(e_{h}+e_{l}\right)b-3\left(1-e_{l}\right)p_{c}t)}{4M}$ Therefore, when $G<G_{1}$, $\frac{\partial \Pi_{mh}^{T*}}{\partial p_{c}}<0$; when $G>G_{1}$, $\frac{\partial \Pi_{mh}^{T*}}{\partial p_{c}}>0$. When $G<G_{2}$, $\frac{\partial \Pi_{ml}^{T/I*}}{\partial p_{c}}<0$; when $G>G_{2}$, $\frac{\partial \Pi_{ml}^{T/I*}}{\partial p_{c}}>0$. When $G<G_{3}$, $\frac{\partial \Pi_{mh}^{I*}}{\partial p_{c}}<0$; when $G>G_{3}$, $\frac{\partial \Pi_{mh}^{I*}}{\partial p_{c}}>0$. □

**Proof of Proposition** **3.** Comparing the profits of the manufacturer in the implementation scenario with that in the transparency scenario, we have

$$\begin{array}{cc} \Pi_{ml}^{I*}-\Pi_{ml}^{T*} & =0\\ \Pi_{mh}^{I*}-\Pi_{mh}^{T*} & =\frac{{\left(a+be_{h}-p_{c}t\left(1-e_{h}\right)\right)}^{2}-{\left(M-p_{c}t\left(e_{h}-e_{l}\right)\right)}^{2}}{8}+Gp_{c}-\frac{{\left[a+be_{h}-p_{c}t\left(1-e_{h}\right)\right]}^{2}}{8}-Gp_{c}\\ \; & =-\frac{{\left(M-p_{c}t\left(e_{h}-e_{l}\right)\right)}^{2}}{8}<0. \end{array}$$

□

**Proof of Proposition** **4.** First, we calculate the manufacturer’s emission reduction advantage under three scenarios.

$$\begin{array}{cc} \Delta^{T}=\Pi_{mh}^{T*}-\Pi_{ml}^{T*} & =\frac{{\left[a+be_{h}-p_{c}t\left(1-e_{h}\right)\right]}^{2}}{8}-\frac{{\left[a+be_{l}-p_{c}t\left(1-e_{l}\right)\right]}^{2}}{8}\\ \Delta^{I}=\Pi_{mh}^{I*}-\Pi_{ml}^{I*} & =\frac{{\left(a+be_{h}-p_{c}t\left(1-e_{h}\right)\right)}^{2}-{\left(M-p_{c}t\left(e_{h}-e_{l}\right)\right)}^{2}}{8}-\frac{{\left(a+be_{l}-p_{c}t\left(1-e_{l}\right)\right)}^{2}}{8}\\ \; & =\Delta^{T}-\frac{{\left(M-p_{c}t\left(e_{h}-e_{l}\right)\right)}^{2}}{8}\\ \Delta^{O}=\Pi_{mh}^{O*}-\Pi_{ml}^{O*} & =0 \end{array}$$

Then, we can easily get

$$\Delta^{T}>\Delta^{I}>\Delta^{O}$$

□

**Proof of Proposition** **5.** Comparing the quantity and profits of the retailer in the implementation scenario with that in the transparency scenario, we have

$$\begin{array}{cc} q_{l}^{I*}-q_{l}^{T*} & =0\\ q_{h}^{I*}-q_{h}^{T*} & =\frac{a+be_{h}-p_{c}t\left(1-e_{l}\right)+M}{4}-\frac{a+be_{h}-p_{c}t\left(1-e_{h}\right)}{4}\\ \; & =\frac{\sqrt{b\left(e_{h}-e_{l}\right)\left(2a+be_{h}+be_{l}-2p_{c}t+2e_{l}p_{c}t\right)}-p_{c}t\left(e_{h}-e_{l}\right)}{4}<0\\ \Pi_{rl}^{I*}-\Pi_{rl}^{T*} & =0\\ \Pi_{rh}^{I*}-\Pi_{rh}^{T*} & ={\left(q_{h}^{I*}\right)}^{2}-{\left(q_{h}^{T*}\right)}^{2}=\left(q_{h}^{I*}+q_{h}^{T*}\right)\left(q_{h}^{I*}-q_{h}^{T*}\right)<0 \end{array}$$

□

**Proof of Corollary** **2.** First, we find the derivative of the optimal wholesale price with respect to the consumer environmental awareness under various scenarios.

$$\begin{array}{cc} \; & \frac{\partial w_{i}^{T*}}{\partial b}=\frac{e_{i}}{2}>0\\ \; & \frac{\partial w_{l}^{I*}}{\partial b}=\frac{e_{l}}{2}>0\\ \; & \frac{\partial w_{h}^{I*}}{\partial b}=\frac{e_{h}M-\left(e_{h}-e_{l}\right)\left(a+b\left(e_{h}+e_{l}\right)-p_{c}t\left(1-e_{l}\right)\right)}{2M}\\ \; & \frac{\partial w_{l}^{O*}}{\partial b}=\frac{e_{l}}{2}>0\\ \; & \frac{\partial w_{h}^{O*}}{\partial b}=\frac{e_{h}\sqrt{b\left(e_{h}-e_{l}\right)\left(2a+be_{h}+be_{l}\right)}-\left(e_{h}-e_{l}\right)\left(a+b\left(e_{h}+e_{l}\right)\right)}{2\sqrt{b\left(e_{h}-e_{l}\right)\left(2a+be_{h}+be_{l}\right)}} \end{array}$$

Let $\frac{\partial w_{h}^{I*}}{\partial b}=0$, so we can get $b=\frac{\left(e_{h}-e_{l}\right)\left(a-p_{c}t\left(1-e_{l}\right)\right)}{e_{l}\left(e_{h}+e_{l}\right)}$. So, when $b<\frac{\left(e_{h}-e_{l}\right)\left(a-p_{c}t\left(1-e_{l}\right)\right)}{e_{l}\left(e_{h}+e_{l}\right)}$, $\frac{\partial w_{h}^{I*}}{\partial b}<0$; when $b>\frac{\left(e_{h}-e_{l}\right)\left(a-p_{c}t\left(1-e_{l}\right)\right)}{e_{l}\left(e_{h}+e_{l}\right)}$, $\frac{\partial w_{h}^{I*}}{\partial b}>0$. Moreover, let $\frac{\partial w_{h}^{O*}}{\partial b}=0$, and we can get $b=\frac{a(e_{h}-e_{l})}{e_{l}\left(e_{h}+e_{l}\right)}$. So, when $b<\frac{a(e_{h}-e_{l})}{e_{l}\left(e_{h}+e_{l}\right)}$, $\frac{\partial w_{h}^{O*}}{\partial b}<0$; when $b>\frac{a(e_{h}-e_{l})}{e_{l}\left(e_{h}+e_{l}\right)}$, $\frac{\partial w_{h}^{O*}}{\partial b}>0$. Then, we find the derivative of the optimal quantity with respect to the consumer environmental awareness under various scenarios.

$$\begin{array}{cc} \; & \frac{\partial q_{i}^{T*}}{\partial b}=\frac{e_{i}}{2}>0\\ \; & \frac{\partial q_{l}^{I*}}{\partial b}=\frac{e_{l}}{2}>0\\ \; & \frac{\partial q_{h}^{I*}}{\partial b}=\frac{e_{h}M+2\left(e_{h}-e_{l}\right)\left(a+b\left(e_{h}+e_{l}\right)-p_{c}t\left(1-e_{l}\right)\right)}{2M}>0\\ \; & \frac{\partial q_{l}^{O*}}{\partial b}=\frac{e_{l}}{2}>0\\ \; & \frac{\partial q_{h}^{O*}}{\partial b}=\frac{e_{h}\sqrt{b\left(e_{h}-e_{l}\right)\left(2a+be_{h}+be_{l}\right)}+\left(e_{h}-e_{l}\right)\left(a+b\left(e_{h}+e_{l}\right)\right)}{2\sqrt{b\left(e_{h}-e_{l}\right)\left(2a+be_{h}+be_{l}\right)}}>0 \end{array}$$

Moreover, we can get

$$\frac{\partial \Pi_{ri}^{*}}{\partial b}=\frac{\partial {\left(q_{i}^{*}\right)}^{2}}{\partial b}=2q_{i}^{*}\frac{\partial q_{i}^{*}}{\partial b}>0$$

Finally, we find the derivative of the optimal manufacturer profit, with respect to the consumer environmental awareness under various scenarios.

$$\begin{array}{cc} \; & \frac{\partial \Pi_{mi}^{T*}}{\partial b}=\frac{\left(a+be_{i}-p_{c}t\left(1-e_{i}\right)\right)e_{i}}{4}>0\\ \; & \frac{\partial \Pi_{ml}^{I*}}{\partial b}=\frac{\left(a+be_{l}-p_{c}t\left(1-e_{l}\right)\right)e_{l}}{4}>0\\ \; & \frac{\partial \Pi_{mh}^{I*}}{\partial b}=\frac{p_{c}t{\left(e_{h}-e_{l}\right)}^{2}\left(a+be_{l}-p_{c}t\left(1-e_{l}\right)+be_{h}\right)+M\left(ae_{l}+be_{l}^{2}+pc_{t}\left(e_{h}^{2}+e_{l}^{2}-e_{l}-e_{l}e_{h}\right)\right)}{4M}>0\\ \; & \frac{\partial \Pi_{ml}^{O*}}{\partial b}=\frac{\partial \Pi_{mh}^{O*}}{\partial b}=\frac{\left(a+be_{l}\right)e_{l}}{4}>0 \end{array}$$

□

## Appendix B. Proofs

### Appendix B.1. Pooling Equilibrium

Under the pooling equilibrium, not inferring the type of manufacturer, the retailer decides the quantity according to its expected profit:

$$q_{p}^{*}\left(w_{p}\right)=\arg\max_{q_{p}}\left[a-q_{i}+b\left(\mu e_{h}+\left(1-\mu \right)e_{l}\right)-w_{p}\right]q_{p}=\frac{a+b\left(\mu e_{h}+\left(1-\mu \right)e_{l}\right)-w_{p}}{2}.$$

So, the *i*-type manufacturer’s objective function is

$V_{ip}=\frac{\left(w_{p}-\left(1-e_{i}\right)p_{c}t\right)\left(a+b\left(\mu e_{h}+\left(1-\mu \right)e_{l}\right)-w_{p}\right)}{2}+Gp_{c}$ and the following optimization problem needs to be solved:

(A4) $$\left\{\begin{array}{c} \max_{w_{p}}V_{hp}\left(w_{p}\right),\\ \max_{w_{p}}V_{lp}\left(w_{p}\right), \end{array}\right.$$

s.t.

(A5) $$V_{hp}\left(w_{p}\right)\geq V_{hd}^{*},$$

(A6) $$V_{lp}\left(w_{p}\right)\geq V_{ld}^{*},$$

where $V_{hd}^{*}=\max\frac{\left(w_{p}-\left(1-e_{h}\right)p_{c}t\right)\left(a+be_{l}-w_{p}\right)}{2}+Gp_{c}=\frac{{\left(a+be_{l}-\left(1-e_{h}\right)p_{c}t\right)}^{2}}{8}+Gp_{c}$ and $V_{ld}^{*}=\max\frac{\left(w_{p}-\left(1-e_{l}\right)p_{c}t\right)\left(a+be_{l}-w_{p}\right)}{2}+Gp_{c}=\frac{{\left(a+be_{l}-\left(1-e_{l}\right)p_{c}t\right)}^{2}}{8}+Gp_{c}$, which represent the maximum profits of the high-type and low-type manufacturer, respectively, that enable the retailer to infer that the carbon emission reduction is low. Constraints (A5) and (A6) limit whether the manufacturer will decide on the same wholesale price, rather than on a deviated wholesale price, to make the retailer infer that he is low type.

Firstly, considering unconstrained optimization problems, i.e., problems (A4), the first-best solutions of the two type manufacturer are $w_{p}^{l*}=\frac{a+\left(1-e_{l}\right)p_{c}t+b\left(\mu e_{h}+\left(1-\mu \right)e_{l}\right)}{2}$ and $w_{h}^{h*}=\frac{a+\left(1-e_{h}\right)p_{c}t+b\left(\mu e_{h}+\left(1-\mu \right)e_{l}\right)}{2}$. The equilibrium wholesale price is the smaller of the two solutions:

(A7) $$w_{p}^{*}=\min\left(w_{p}^{l*},w_{p}^{h*}\right)=w_{p}^{h*}.$$

For the high-type manufacturer, pooling at a wholesale price higher than $w_{p}^{h*}$ is always inferior to deciding $w_{p}^{h*}$ for any reasonable beliefs. Thus, the low-type manufacturer has to compromise on the same wholesale price $w_{p}^{h*}$. Consequently, to achieve pooling equilibrium, the maximum wholesale price is $w_{p}^{h*}$.

Second, solving inequality (A5) and (A6), we can obtain that $w_{p}$ belongs to the following intervals:

$$w_{p}\in \left[w_{p1},w_{p2}\right]\cap \left[w_{p3},w_{p4}\right],$$

where $w_{p1}=\frac{a+\left(1-e_{h}\right)p_{c}t+b\left(\mu e_{h}+\left(1-\mu \right)e_{l}\right)-\sqrt{b\mu \left(e_{h}-e_{l}\right)\left(2a+b\left(\mu e_{h}+2e_{l}-\mu e_{l}\right)-2\left(1-e_{h}\right)p_{c}t\right)}}{2}$, $w_{p2}=$$\frac{a+\left(1-e_{h}\right)p_{c}t+b\left(\mu e_{h}+\left(1-\mu \right)e_{l}\right)+\sqrt{b\mu \left(e_{h}-e_{l}\right)\left(2a+b\left(\mu e_{h}+2e_{l}-\mu e_{l}\right)-2\left(1-e_{h}\right)p_{c}t\right)}}{2}$, $w_{p3}=$$\frac{a+\left(1-e_{l}\right)p_{c}t+b\left(\mu e_{h}+\left(1-\mu \right)e_{l}\right)-\sqrt{b\mu \left(e_{h}-e_{l}\right)\left(2a+b\left(\mu e_{h}+2e_{l}-\mu e_{l}\right)-2\left(1-e_{l}\right)p_{c}t\right)}}{2}$ and $w_{p4}=$$\frac{a+\left(1-e_{l}\right)p_{c}t+b\left(\mu e_{h}+\left(1-\mu \right)e_{l}\right)+\sqrt{b\mu \left(e_{h}-e_{l}\right)\left(2a+b\left(\mu e_{h}+2e_{l}-\mu e_{l}\right)-2\left(1-e_{l}\right)p_{c}t\right)}}{2}$. Then, we can easily get $w_{p1}<w_{p3}$,$w_{p1}<w_{p}^{*}<w_{p2}$ and $w_{p3}<w_{p}^{*}<w_{p4}$ which verify that $w_{p}^{*}$ is the optimal solution without deviation, and the minimum value of pooling is $w_{p3}$.

All in all, under the pooling equilibrium, the manufacturer’s optimal wholesale price and the retailer’s belief structure are

$$\begin{array}{cc} \; & w_{p}^{*}=\frac{a+\left(1-e_{h}\right)p_{c}t+b\left(\mu e_{h}+\left(1-\mu \right)e_{l}\right)}{2},\\ \; & \mathrm{Pr}\left(\tilde{e}=e_{h}\right)=\left\{\begin{array}{cc} 1 & if\;w<w_{p3},\\ \mu & if\;w_{p3}\leq w\leq w_{p}^{*},\\ 0 & if\;w>w_{p}^{*}. \end{array}\right. \end{array}$$

### Appendix B.2. The Intuitive Criterion Refinement

The intuitive criterion verifies the equilibrium between the signal sender and signal receiver in two steps.

(1) Step 1. Find a set Θ of the types of signal sender in which the payoff under equilibrium strategy is greater than the maximum payoff deviating from this equilibrium. In this case, the off-equilibrium strategy is dominated by the equilibrium strategy, that is, the *i*-type sender where i∈Θ will never choose the deviation strategy. If the set Θ contains all types of sender, the refinement ends and the equilibrium satisfies the intuitive criterion. If not, proceed to step 2.

Specifically, in our model, we first find the type set Θ in which the *i*-type manufacturer meets the following condition

(A8) $$V_{i}^{*}>{\hat{V}}_{i}\left(w\right),$$

where $V_{i}^{*}$ is the *i*-type manufacturer’s equilibrium profit and $\hat{V}\left(w\right)$ is the maximum deviation profit when the manufacturer determines an off-equilibrium wholesale price *w*. In all retailers’ beliefs, only when she infers that the carbon emission reduction is high, the manufacturer can obtain the maximum profit

$$\hat{V}\left(w\right)=\frac{\left(w-\left(1-e_{i}\right)p_{c}t\right)\left(a+be_{h}-w\right)}{2}+Gp_{c}.$$

Then, we analyze the types in the set Θ to decide whether to go to the next step.

(2) Step 2. We first define the set $\Theta^{c}$ as the complement of set Θ. Such an inference can be made that any off-equilibrium signal is determined by the *j*-type sender where $j\in \Theta^{c}$, because the *i*-type sender will never deviate. Then, under this inference, we check the set $\Theta^{c}$ in which the payoff under equilibrium strategy is not less than the minimum payoff deviating from this equilibrium. If all types of signal senders in the set $\Theta^{c}$ meet the condition, the intuitive criterion is passed.

Specifically, in our model, we check the type set $\Theta^{c}$ in which *j*-type manufacturer meets the following condition

(A9) $$V_{j}^{*}>{\tilde{V}}_{j}\left(w\right),$$

where $V_{j}^{*}$ is *j*-type manufacturer’s equilibrium profit and ${\tilde{V}}_{j}\left(w\right)$ is the minimum deviation profit when the manufacturer determines an off-equilibrium wholesale price *w*. According to the types contained in the set $\Theta^{c}$, there are two cases of the minimum deviation profit:

$${\tilde{V}}_{j}\left(w\right)=\left\{\begin{array}{cc} \frac{\left(w-\left(1-e_{j}\right)p_{c}t\right)\left(a+be_{l}-w\right)}{2}+Gp_{c} & if\;l\in \Theta^{c},\\ \frac{\left(w-\left(1-e_{j}\right)p_{c}t\right)\left(a+be_{h}-w\right)}{2}+Gp_{c} & \mathrm{otherwise}. \end{array}\right.$$

If all types of signal senders in the set $\Theta^{c}$ meet the condition, the equilibrium survives the intuitive criterion.

### Appendix B.3. The Elimination of Pooling Equilibrium

We can always find $w_{1}<w_{p}^{*}$ such that $V_{hh}\left(w_{1}\right)=V_{hp}\left(w_{p}^{*}\right)$

$$V_{hh}\left(w_{1}\right)=\frac{\left(w_{1}-\left(1-e_{h}\right)p_{c}t\right)\left(a+be_{h}-w_{1}\right)}{2}+Gp_{c},$$

$$V_{hp}\left(w_{p}^{*}\right)=\frac{\left(w_{p}^{*}-\left(1-e_{h}\right)p_{c}t\right)\left(a+b\left(\mu e+\left(1-\mu \right)e_{l}\right)-w_{p}^{*}\right)}{2}+Gp_{c}.$$

Then, we substitute $w_{1}$ and $w_{p}^{*}$ into $V_{lh}\left(w\right)$ and $V_{lp}\left(w\right)$, and get

$$V_{lh}\left(w_{1}\right)=\frac{\left(w_{1}-\left(1-e_{l}\right)p_{c}t\right)\left(a+be_{h}-w_{1}\right)}{2}+Gp_{c},$$

$$V_{lp}\left(w_{p}^{*}\right)=\frac{\left(w_{p}^{*}-\left(1-e_{l}\right)p_{c}t\right)\left(a+b\left(\mu e_{h}+\left(1-\mu \right)e_{l}\right)-w_{p}^{*}\right)}{2}+Gp_{c}.$$

So,

$$V_{hh}\left(w_{1}\right)-V_{lh}\left(w_{1}\right)=\frac{\left(e_{h}-e_{l}\right)p_{c}t\left(a+be_{h}-w_{1}\right)}{2},$$

$$V_{hp}\left(w_{p}^{*}\right)-V_{lp}\left(w_{p}^{*}\right)=\frac{\left(e_{h}-e_{l}\right)p_{c}t\left(a+b\left(\mu e_{h}+\left(1-\mu \right)e_{l}\right)-w_{p}^{*}\right)}{2}.$$

For $w_{1}<w_{p}^{*}$ and be>bμe, it is easy to obtain

$$V_{hh}\left(w_{1}\right)-V_{lh}\left(w_{1}\right)>V_{hp}\left(w_{p}^{*}\right)-V_{lp}\left(w_{p}^{*}\right).$$

As mentioned earlier, $V_{hh}\left(w_{1}\right)=V_{hp}\left(w_{p}^{*}\right)$, so

$$V_{lh}\left(w_{1}\right)<V_{lp}\left(w_{p}^{*}\right).$$

So, as shown in Figure A1, we can find $w_{2}=w_{1}+\epsilon$ where ϵ>0 and it is small enough, satisfying the following inequality

$$V_{hp}\left(w_{p}^{*}\right)<V_{hh}\left(w_{2}\right),$$

$$V_{lp}\left(w_{p}^{*}\right)>V_{lh}\left(w_{2}\right).$$

Therefore, when the manufacturer determines an off-equilibrium wholesale price $w_{2}$, we can find Θ=l. So, for *h*-type manufacturer, the minimum deviation profit is $V_{hh}\left(w_{2}\right)$ but $V_{hp}\left(w_{p}^{*}\right)<V_{hh}\left(w_{2}\right)$. So, the pooling equilibrium violates the intuitive criterion. In other words, under the inference that a deviation to $w_{2}$ makes the retailer believe the manufacturer is a high type, the high-type manufacturer is willing to deviate from $w_{p}^{*}$ to $w_{2}$, but the low-type manufacturer keeps $w_{p}^{*}$ unchanged. Thus, the two types are separated.
